# Bioremediation of distillery effluent by *Pleurotus sajor-caju*: evaluation of the influence of pH in vinasse derived from molasses

**DOI:** 10.1186/1753-6561-8-S4-P190

**Published:** 2014-10-01

**Authors:** Moniky Aragão, Diego Menezes, Helon Oliveira, Jaqueline Souza, Osiris Vital-Brazil, Luiz Romanholo-Ferreira, Regina Monteiro, Ana Queijeiro-Lopez, José Teixeira, Maria Hernandez-Macedo, Denise Ruzene, Daniel Silva

**Affiliations:** 1 Universidade Tiradentes, Instituto de Tecnologia e Pesquisa, Aracaju, SE, Brasil; 2 Universidade de São Paulo, Centro de Energia Nuclear na Agricultura, Piracicaba, SP, Brasil; 3 Universidade Federal de Alagoas, Instituto de Química e Biotecnologia, Maceió, AL, Brasil; 4 IBB/Uminho, Inst. de Biotecnologia e Bioengenharia, Universidade do Minho, Braga, Portugal; 5 Universidade Federal de Sergipe, Núcleo de Engenharia de Produção, São Cristovão, SE, Brasil

## Background

The alcohol industry is an excellent representation of the development process in Brazil, it considers the sugar cane as one of the biggest monocultures. As a result of this high production, there is the vinasse, a residue from the alcohol production after the pulp fermentation and distillation of the wine (Pulp after fermentation), ensuing from 8 to 15 liters for each liter of ethanol produced. This high colored effluent and an objectionable odor is rich in nutrients, mainly in organic matter, having a high potencial poluent when it is inaccordingly in the environment [1,2]. Lignolytic fungi can be used to the remediation of the pollutants, such as vinasse, by the action of peroxidases. So, the search is interested in reducing the contaminations caused by the direct application of the vinasse on the earth without a previous treatment. Therefore the objective is to characterize the biodegradation of the residue through the basidiomycetes *Pleurotus sajor-cajuCCB 020 *and consequently to produce enzymes with biotechnological potential, decoloring the vinasse and minimizing the polluter potential. After the treatment of the vinasse, it shows several goals as the fertirrigation, the water reuse for the washing proccess of the sugar cane and/or other activities related to the industrial process.

## Methods

The fungi were inoculated in the vinasse which had its pH previously corrected to 4.0 e 6.0 using solutions of NaOH (2.5 M) and/or HCl (5.0 M), during a period of 16 days at 82.4º F in agitation of 180 rpm with no light. The control consisted in material without the fungi inoculation and in the synthetic environment MSF. After it was realized 7 collections to determine the pH variation and the decolorization factor according to the formula used by Itoh (2005) [3] and Sirianuntapiboon et al. (1995) [4] from the absorbance standart at 475 nm: Decoloration (%) = [(absorbance initial - absorbance observed) / absorbance initial]. Besides these parameters, the production taxes of the fungi biomass were evaluated. All the reactions were realized in triplicate and the lectures of the absorbance made with the spectrophotometer support FEMTO 432.

## Results and conclusion

According to the results, the presence of different levels in the vinasse decoloration which was concomitant with the toxicity decrease, and the production increase of the fungi biomass. However these parameters were more satisfactory when they were at 82.4ºF and pH 6.0, reaching an utmost of 90% of decoloration and a value of biomass concentration equivalent to 11.07g/L. Therefore it is concluded that the *P. sajor-caju *is able in the applicability of the color remotion and in the degradation of the vinasse toxic compounds from the sugar cane, it can be reused after the treatment in biotechnological process aggregating values to a subproduct that causes environment problems. These factors indicate a possible and promissor process of use of residues in a viable way to industrial objectives.
